# Clinical efficacy of argon plasma coagulation combined with cryotherapy for central airway stenosis caused by lung cancer

**DOI:** 10.1186/s13019-019-0979-7

**Published:** 2019-08-28

**Authors:** Zhiguo Wang, Wei Wang, Guocheng Wu

**Affiliations:** Department of Respiratory and Critical Care Medicine, The PLA Navy Anqing Hospital, Anqing, 246003 Anhui Province China

**Keywords:** Argon plasma coagulation, Cryotherapy, Central airway stenosis

## Abstract

**Background:**

This study aimed to study the clinical efficacy of argon plasma coagulation plus cryotherapy for central airway stenosis caused by lung cancer.

**Methods:**

The clinical data of 177 patients with central airway stenosis caused by lung cancer received surgery from June, 2017 to July 2018 were retrospectively analyzed. According to different treatments, 43 patients received cryotherapy were included in the control group, while 134 patients received argon plasma coagulation plus cryotherapy were in the observation group. After propensity score matching, patients in the two groups were in a 1:1 ratio. The Karnofsky score, partial pressure of oxygen (PaO_2_), arterial oxygen saturation (SaO_2_), partial pressure of arterial carbon dioxide (PaCO_2_) and adverse reactions in patients were analyzed one week before and after treatment. Besides, the survival rates of the two groups were compared.

**Results:**

After propensity score matching, the baseline data were not significantly different between the two groups. The post-treatment Karnofsky scores in the two groups were significantly higher than those of before treatment, and the post-treatment score of the observation group was higher than that of the control group (all *P* < 0.05). The post-treatment PaO_2_ and SaO_2_ in the observation group were both higher than those of the control group; while the PaCO_2_ in the observation group was significantly lower than that of the control group (all *P* < 0.05). In the observation group, the levels of PaO_2_ and SaO_2_ were significantly higher, and the level of PaCO_2_ was significantly lower after treatment than those of before treatment (all *P* < 0.05). The rates of completely effective and mild effective in the observation group were significantly higher than those in the control group (both *P* < 0.05). The incidences of bleeding, arrhythmia and fever in the observation group were significantly lower than those in the control group (all *P* < 0.05). The survival rate was significantly higher in the observation group (72.09%) than in the control group (51.16%).

**Conclusions:**

Argon plasma coagulation combined with cryotherapy can significantly alleviate the central airway stenosis caused by lung cancer, reduce the incidence of adverse reactions, and improve prognosis in patients.

## Background

Lung cancer is currently with a high clinical incidence, and most patients did not realize the cancer until advanced stage due to its atypical symptoms. At this stage, patients may suffer symptoms such as central airway stenosis, shortness of breath, hemoptysis, and cough [1]. Chemotherapy can not reduce the size of tumor tissues in the trachea in a short period of time, and may induce edema, further aggravating airway obstruction [2]. Traditional surgery can lead to large trauma on patients, so some patients cannot directly undergo surgery because of their poor physical condition [3]. Cryotherapy refers to the use of liquid gas (such as nitrogen or carbon dioxide) to freeze the lesion tissues, resulting in damage and necrosis in structure under low temperature, which makes lesions easy to remove [4]. The argon plasma coagulation performs non-contact thermocoagulation on the lesions by ionizing argon gas, thereby clearing the lesion tissues [5]. The dyspnea index refers the respiratory state of the patient, and higher level indicates worse respiratory state [6]. The Karnofsky score is a measure of overall health, and increase in the score indicates health improvement in the patient [7]. Study in 47 patients with tumor-induced central airway stenosis treated by argon plasma coagulation plus cryotherapy showed improved or disappeared symptoms such as dyspnea, cough, and hemoptysis, as well as significantly improved respiratory function in patients [8]. Nevertheless, cryotherapy can poorly clear major tumor tissues, and easily cause bleeding from the wound during operation. The argon plasma coagulation damages the lesion tissues through thermal effect, which may injure patient’s tracheal mucosa in a certain degree [9]. In this study, argon plasma coagulation combined with cryotherapy were used to treat central airway stenosis caused by lung cancer, so as to analyze the effect of the combination on central airway stenosis.

## Materials and methods

### Subjects

Clinical data of 177 patients with central airway stenosis caused by lung cancer who underwent surgery from June 2017 to July 2018 in The PLA Navy Anqing Hospital were retrospectively analyzed. According to different treatment method, 43 patients received cryotherapy were included in the control group, while 134 patients received argon plasma coagulation plus cryotherapy were included in the observation group. Propensity score matching was used for further screening, so that the patients in the two groups were in a 1:1 ratio, with 43 cases in each group. After propensity score matching, there were 26 males and 17 females in the control group, aged 36–75 years, with an average age of 46.7 ± 5.3 years; in the observation group, there were 23 males and 20 females, aged 32–69 years, with an average age of 47.4 ± 6.1 years. Patients were eligible if they had lung cancer at stage II-IV; had poor physical condition and cannot undergo surgical resection; and did not received bronchoscopy intervention for central airway stenosis before. Patients were excluded if they had central airway stenosis caused by non-lung cancer; had immune diseases, organ failure, other malignant tumors, or mental instability; were allergic to treatment drugs, or in pregnancy; had incomplete baseline or outcome data; were uncooperative. This study was approved by the Ethics Committee of The PLA Navy Anqing Hospital, and informed consent was obtained from all the subjects.

### Therapeutic method

Patients in the two groups were all treated with radiotherapy, chemotherapy or targeted therapy (at least one of them). In case of intestinal reaction, antiemetic drugs were prescribed to relieve the symptoms, and gastric mucosa was protected by using gastrointestinal mucosa protectors and proton pump inhibitors. Patients went through enhanced computed tomography, electrocardiogram, blood gas analysis, and strict bronchoscopy for clinical information collection. Before surgery, they were fasted for about 3 h, and given phenobarbital (Tianjin KingYork, China) for sedation, and 2% lidocaine (Shanghai Zhpharma, China) for spraying anesthesia on their mouth and nose. An appropriate amount of fentanyl (Yichang Humanwell, China) can be injected intravenously according to patient’s condition.

Then, patients in the control group were treated with cryotherapy. The cryoprobe was acted on the lesion tissues at − 70 °C to − 45 °C through the hole of bronchoscopy biopsy, and the frozen lesion was excised after congelation. Then, a stent was placed to keep the airway open. Patients in the observation group were treated with argon plasma coagulation plus cryoablation. The sequence of the two treatments may vary according to patient’s condition. The front end of the injection duct of argon was reached out along the bronchoscope until 0.5 cm away from the tumor tissue. Then, the multi-point coagulation was performed according to the range of lesion tissues. The coagulation time was no more than 3 s, and the eschar on the surface of the lesions was cleared in time.

### Outcome measures

The partial pressure of oxygen (PaO_2_), arterial oxygen saturation (SaO_2_), partial pressure of arterial carbon dioxide (PaCO_2_) were detected one week before and after the treatment, also the occurrences of bleeding, arrhythmia, fever, and hypoxia (PaO_2_ less than 60 mmHg) during the postoperative week were recorded. The Karnofsky score in patients was evaluated according to patients’ activity ability, self-care ability and disease level. The higher the score, the better the health condition of the subject, and 100 points indicated a healthy condition; 0 point indicated a critical state. The dyspnea index in patients was also measured, and grade 0 referred to eupnea; grade 1 referred to shortness of breath during walking; grade 2 referred to shortness of breath during fast walking; grade 3 referred unable to walk because of shortness of breath; grade 4 referred extremely easy occurrence of shortness of breath [6].

### Evaluation criteria

The efficacy was evaluated one week after treatment, and the criteria of effect on airway stenosis were as follows: completely effective referred to complete clear of airway obstruction and normal respiratory function; partially effective referred to over 50% reduction of airway obstruction and improved respiratory function; mild effective referred to 20–50% reduction of airway obstruction and partially improved respiratory function; ineffective referred to no improvement in airway obstruction [8].

The follow up was carried out through outpatient or telephone until May 31, 2019 to record the clinical symptoms and survival.

### Statistical analyses

Data in this study were processed with the use of SPSS.21.0. To reduce selection bias, propensity score matching was used for further subject screening, so that the patients in the two groups were in a 1:1 ratio. The measurement data were expressed as mean ± standard deviation, and processed using paired t test (between before and after intervention within group) and independent sample t test (between groups at the same time point). The count data were expressed as rate, processed using χ^2^ test. Ranked data were processed using Wilcoxon-Mann-Whitney test. *P* < 0.05 was considered statistically significant.

## Results

### Analysis of baseline data

At baseline, there were statistically significant differences in BMI, nutritional status, airway obstruction and concomitant hypertension between the observation group and the control group (all *P* < 0.05), while there was no significant difference in gender, age, site of airway obstruction, pathological type, concomitant anemia, or tumor-node-metastasis (TNM) stage between the two groups (all *P* > 0.05). See Table 1. After propensity score matching, the differences in gender, age, body mass index, nutritional status, airway obstruction, site of airway obstruction, pathological type, concomitant disease, and TNM staging were all not significant between the two groups (all *P* > 0.05). See Table 2.

**Table 1:** Clinical data

Group	Control group (*n* = 43)	Observation group (*n* = 134)	t/χ^2^	*P*
Gender (male / female)	26/17	73/61	0.668	0.491
Age (year)	46.7 ± 5.3	45.2 ± 4.8	1.739	0.084
Weight (kg)	57.37 ± 6.72	58.51 ± 7.29	0.909	0.365
BMI (kg/m^2^)	23.28 ± 3.83	21.36 ± 4.32	2.604	0.010
Nutritional status (n)			1.801	0.072
Good	16	63		
Poor	27	71		
Airway obstruction (n)			2.879	0.004
Mild	14	31		
Severe	29	103		
Site of airway obstruction (n)			0.505	0.613
Trachea	18	62		
Right and left main bronchus	25	72		
Pathological type (n)			0.513	0.736
Squamous cell carcinoma	11	28		
Adenocarcinoma	15	37		
Large cell carcinoma	12	42		
Small cell carcinoma	5	27		
Concomitant disease (n, %)				
Hypertension	13 (30.23%)	65 (48.51%)	2.100	0.036
Anemia	14 (32.56%)	36 (26.87%)	0.721	0.471
TNM staging (n)			0.348	0.625
II	7	21		
III	14	48		
IV	22	65

**Table 2:** Baseline data after propensity score matching

Group	Control group (*n* = 43)	Observation group (*n* = 43)	t/χ^2^	*P*
Sex (male / female)	26/17	23/20	0.653	0.514
Age (year)	46.7 ± 5.3	47.4 ± 6.1	0.518	0.606
Weight (kg)	57.37 ± 6.72	59.21 ± 7.16	1.229	0.223
BMI (kg/m^2^)	23.28 ± 3.83	24.42 ± 4.64	1.423	0.218
Nutritional status (n)			0.684	0.494
Good	16	13		
Poor	27	30		
Airway obstruction (n)			0.453	0.651
Mild	14	16		
Severe	29	27		
Site of airway obstruction (n)			1.841	0.065
Trachea	18	10		
Right and left main bronchus	25	33		
Pathological type (n)			0.434	0.805
Squamous cell carcinoma	11	10		
Adenocarcinoma	15	13		
Large cell carcinoma	12	14		
Small cell carcinoma	5	6		
Concomitant disease (n, %)				
Hypertension	13 (30.23)	12 (27.91)	0.238	0.812
Anemia	14 (32.56)	13 (30.23)	0.232	0.816
TNM staging (n)			0.632	0.729
II	7	9		
III	14	11		
IV	22	23		

### Comparison of airway diameter and dyspnea index

**Fig. 1:**
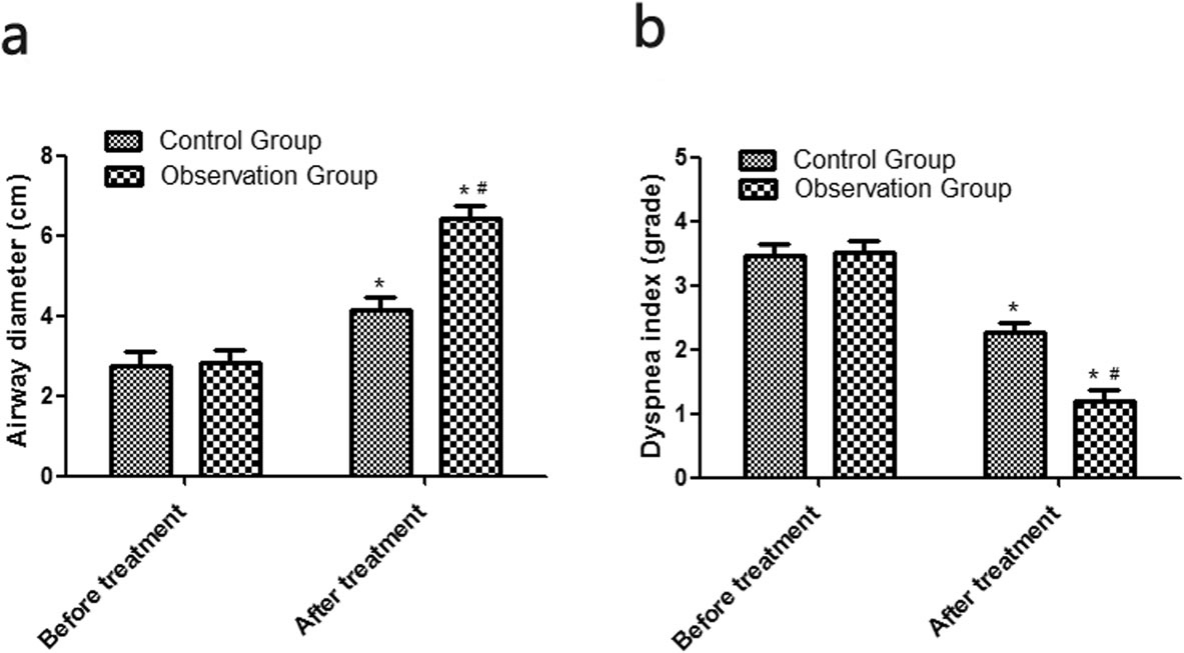
Comparison of airway diameter and dyspnea index. **a** Airway diameter. **b** Dyspnea index. Compared with the control group, ^#^*P* < 0.05; compared with before treatment, ^*^P < 0.05

Before treatment, the airway diameters of the control group and the observation group were 2.68 ± 0.42 cm, and 2.68 ± 0.47 cm, respectively, without statistical difference (*P* > 0.05). The post-treatment airway diameters of the control group and the observation group were 4.29 ± 0.64 cm and 6.34 ± 0.86 cm respectively, which were both significantly larger than those of before treatment, and the post-treatment diameter of the observation group was larger than that of the control group (all *P* < 0.05). Before treatment, the dyspnea index of the control group and the observation group were grade 3.41 ± 0.39 and grade 3.47 ± 0.36, respectively, without statistical difference (*P* > 0.05). The post-treatment dyspnea index of the control group and the observation group were grade 2.46 ± 0.36 and grade 1.29 ± 0.27 respectively, which were significantly lower than those of before treatment, and the post-treatment index of the observation group was lower than that of the control group (all *P* < 0.05). See Fig. 1.

### Comparison of Karnofsky score

**Fig. 2:**
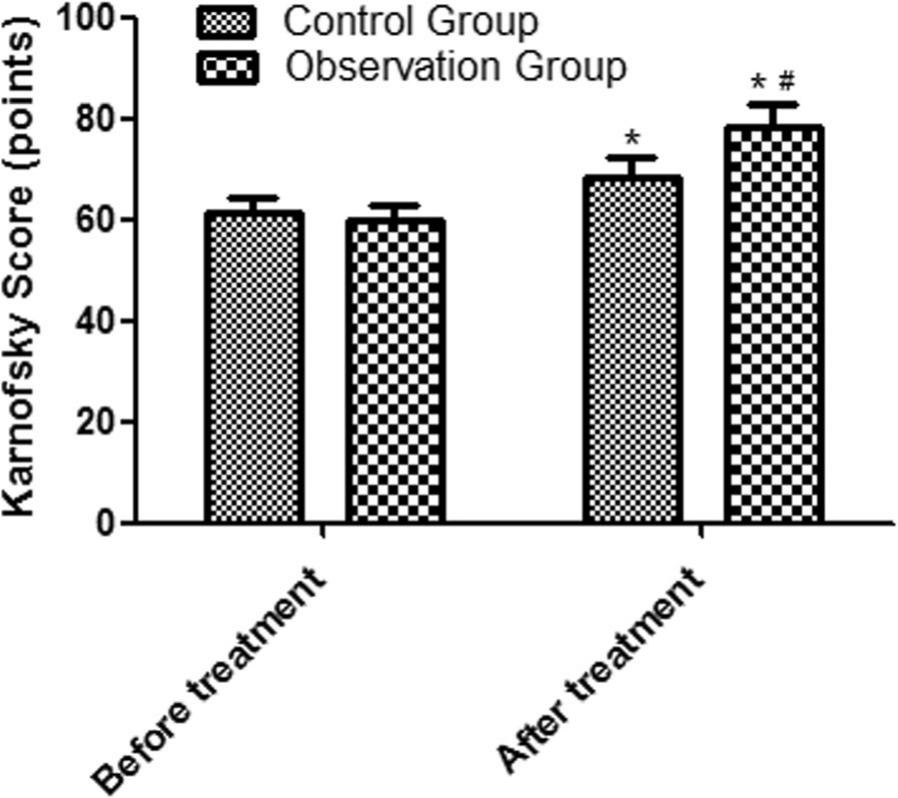
Comparison of Karnofsky score. Compared with the control group, ^#^*P* < 0.05; compared with before treatment, ^*^P < 0.05

The post-treatment Karnofsky scores of the control group and the observation group were 65.28 ± 7.64 points and 76.29 ± 8.37 points, respectively, which were significantly higher than those of before treatment, and the post-treatment score of the observation group was higher than that of the control group (all *P* < 0.05). See Fig. 2.

### Arterial blood-gas analysis

In the observation group, the post-treatment PaO_2_ was 73.29 ± 6.27 mmHg and SaO_2_ was 97.34 ± 9.24%, which were significantly higher than those of the control group, and PaCO_2_ was 34.26 ± 3.17 mmHg, which was significantly lower than that of the control group (all *P* < 0.05). In both groups, the levels of post-treatment PaO_2_ and SaO_2_ were significantly higher than those of before treatment, and the levels of post-treatment PaCO_2_ were significantly lower than those of before treatment (all *P* < 0.05). See Table 3.

**Table 3:** Comparison of arterial blood-gas parameters

Group	Time	PaO_2_ (mmHg)	PaCO_2_ (mmHg)	SaO_2_ (%)
Control group	Before treatment	54.36 ± 4.63	43.46 ± 3.37	82.46 ± 5.73
	After treatment	64.23 ± 4.62^*^	39.24 ± 3.12^*^	91.36 ± 8.64^*^
Observation group	Before treatment	56.37 ± 5.13	44.51 ± 4.42	83.72 ± 5.48
	After treatment	73.29 ± 6.27^*#^	34.26 ± 3.17^*#^	97.34 ± 9.24^*#^

Compared with the control group, ^#^*P* < 0.05; compared with before treatment, ^*^P < 0.05

### Comparison of effective rate

The rate of completely effective in the observation group was 37.21%, and the rate of mildly effective was 11.63%, which were significantly different from those in the control group (both *P* < 0.05). The partially effective and ineffective rates of the observation group were 46.51 and 4.65%, respectively, and there was no significant difference in these two rates as compared with the control group (both *P* > 0.05). See Table 4.

**Table 4:** Comparison of effective rate (n, %)

Group	Completely effective	Partially effective	Mildly effective	Ineffective
Control group (*n* = 43)	7 (16.28)	17 (39.53)	15 (34.88)	4 (9.31)
Observation group (*n* = 43)	16 (37.21)	20 (46.51)	5 (11.63)	2 (4.65)
χ^2^	2.193	0.653	2.553	0.847
*P*	0.028	0.514	0.011	0.392

### Comparison of post-treatment adverse reactions

In the observation group, the incidence of bleeding was 11.63%; of arrhythmia was 13.95%, and of fever was 18.60%, which were significantly lower than those in the control group (all *P* < 0.05). There were 7 patients with PaO_2_ less than 60 mmHg in the observation group, with an hypoxia incidence of 16.28%, which was not significantly different from that in the control group (*P* > 0.05). See Table 5.

**Table 5:** Comparison of adverse reactions

Group	Hypoxia	Bleeding	Arrhythmia	Fever
Control group (*n* = 43)	6 (13.95)	13 (30.23)	14 (32.56)	17 (39.53)
Observation group (*n* = 43)	7 (16.28)	5 (11.63)	6 (13.95)	8 (18.60)
χ^2^	0.301	2.121	2.042	2.137
*P*	0.763	0.034	0.041	0.033

### Comparison of survival rates

Patients in both groups were followed up to May 31, 2019, with a successful follow-up rate of 100%. In the control group, 21 cases died and 22 cases survived, with a survival rate of 51.16%. In the observation group, 12 cases died and 31 cases survived, with a survival rate of 72.09%. The survival rate in the observation group was significantly higher than that in the control group (*P* < 0.05).

## Discussion

Most patients with lung cancer are diagnosed at the advanced stage with poor health condition, which is a bad timing for surgery [10]. Due to the particularity of lung cancer lesions, excessive growth of tumor tissues can induce airway stenosis and obstruction in patients, leading to dyspnea or shock [11]. Chemoradiotherapy can inhibit the growth of tumor tissues, but cannot timely relieve the dyspnea caused by central airway obstruction, so it is not suitable for critical patients [12, 13]. Therefore, exploring a reasonable plan for the treatment of central airway obstruction is of important clinical significance.

This study found that the post-treatment airway diameter, Karnofsky score, and dyspnea index were better in the observation group than those in the control group, suggesting that the treatment for the observation group was better for the patients’ respiratory function and health condition. We also found that the post-treatment levels of PaO_2_ and SaO_2_ in the observation group were higher than those in the control group, and the PaCO_2_ was lower in the observation group than that in the control group, suggesting that argon plasma coagulation combined with cryotherapy can improve the hypoxic state of patients. Cryotherapy uses liquid nitrogen or carbon dioxide to damage the lesion tissues, and induces necrosis of the lesion through low temperature, which is not easy to cause complications such as bronchial perforation. However, the cryotherapy has limited freezing depth to lesion tissues, so it’s not promising for clearing large tumor tissues, and relieving airway stenosis [14, 15]. Argon plasma coagulation can damage the lesion tissues from various angles, which effectively clears the tumor tissues [16]. In the observation group, the completely effective rate and mildly effective rate were 37.21 and 11.63%, respectively, which were significantly different from those of the control group, while the partially effective rate and ineffective rate were 46.51 and 4.65%, respectively, which were not significantly different from the control group, suggesting that the argon plasma coagulation combined with cryotherapy had better treatment effect, which is consistent with previous study [17].

Adverse reactions including bleeding, arrhythmia, fever and hypoxia occurred in some patients in both groups, but the incidences of bleeding, fever and arrhythmia in the observation group were significantly lower than those in the control group. Argon plasma coagulation can easily increase the temperature of patient’s airway and cause damage to the airway mucosa during operation, leading to airway burns and even perforation, and sometimes hypoxemia. Therefore, it is necessary to pay special attention to the coagulation time during operation [18, 19]. Due to the abundant blood vessels in the airway tumor tissue, the blood coagulation effect of cryotherapy on the bleeding tissue is not favorable, and patients are prone to bleeding [20]. Cryotherapy and excision after argon plasma coagulation can better remove the necrotic tissues, and the hemostatic effect of argon is beneficial to alleviating the tissue damage during cryotherapy, which further reduces airway damage and reduce the incidence of complications [21, 22]. In this study, the mortality of the observation group was lower than that of the control group, probably because argon plasma coagulation combined with cryotherapy showed better efficacy for central airway stenosis, and resulted in lower incidence of complications, thereby improving the prognosis in patients. However, the sample size included in this study was insufficient, and the follow-up period we carried out should be prolonged for further analysis of the postoperative outcome, so further studies with improved protocol are needed.

## Conclusions

Argon plasma coagulation combined with cryotherapy can significantly alleviate the central airway stenosis caused by lung cancer, reduce the incidence of adverse reactions, and improve the prognosis in patients.

## Data Availability

The datasets used and/or analysed during the current study are available from the corresponding author on reasonable request.

## Abbreviations

PaCO_2_: partial pressure of arterial carbon dioxide
PaO_2_: partial pressure of oxygen
SaO_2_: arterial oxygen saturation
TNM: tumor-node-metastasis
